# Inositol Hexaphosphate as an Inhibitor and Potential Regulator of p47^phox^ Membrane Anchoring

**DOI:** 10.1021/acs.biochem.4c00117

**Published:** 2024-04-26

**Authors:** Angela M. Develin, Brian Fuglestad

**Affiliations:** † Department of Chemistry, Virginia Commonwealth University, Richmond, Virginia 22384, United States; ‡ Institute for Structural Biology, Drug Discovery and Development, Virginia Commonwealth University, Richmond, Virginia 23219, United States

## Abstract

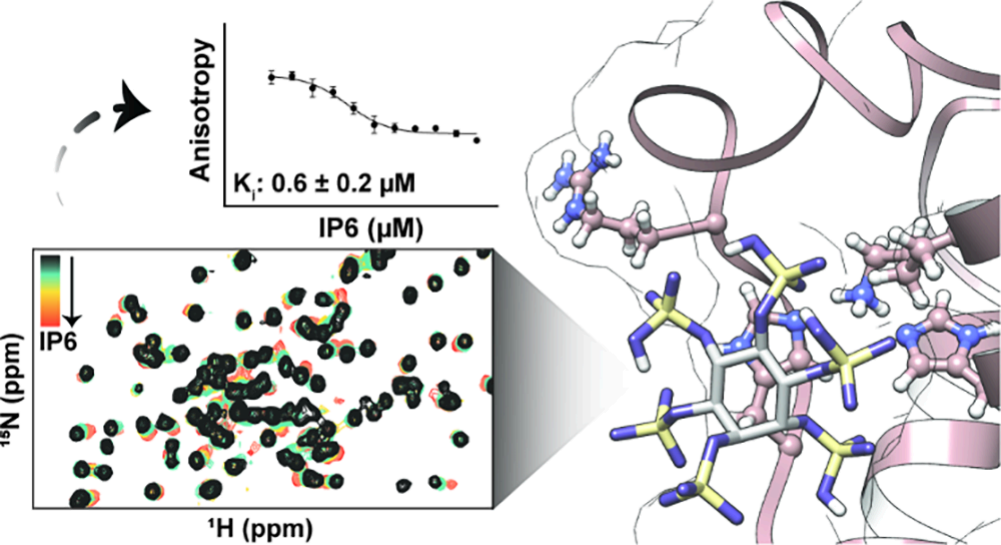

As a key component for NADPH oxidase 2 (NOX2) activation, the peripheral membrane protein p47^phox^ translocates a cytosolic activating complex to the membrane through its PX domain. This study elucidates a potential regulatory mechanism of p47^phox^ recruitment and NOX2 activation by inositol hexaphosphate (IP6). Through NMR, fluorescence polarization, and FRET experimental results, IP6 is shown to be capable of breaking the lipid binding and membrane anchoring events of p47^phox^-PX with low micromolar potency. Other phosphorylated inositol species such as IP5(1,3,4,5,6), IP4(1,3,4,5), and IP3(1,3,4) show weaker binding and no ability to inhibit lipid interactions in physiological concentration ranges. The low micromolar potency of IP6 inhibition of the p47^phox^ membrane anchoring suggests that physiologically relevant concentrations of IP6 serve as regulators, as seen in other membrane anchoring domains. The PX domain of p47^phox^ is known to be promiscuous to a variety of phosphatidylinositol phosphate (PIP) lipids, and this regulation may help target the domain only to the membranes most highly enriched with the highest affinity PIPs, such as the phagosomal membrane, while preventing aberrant binding to other membranes with high and heterogeneous PIP content, such as the plasma membrane. This study provides insight into a potential novel regulatory mechanism behind NOX2 activation and reveals a role for small-molecule regulation in this important NOX2 activator.

## Introduction

**Figure 1:**
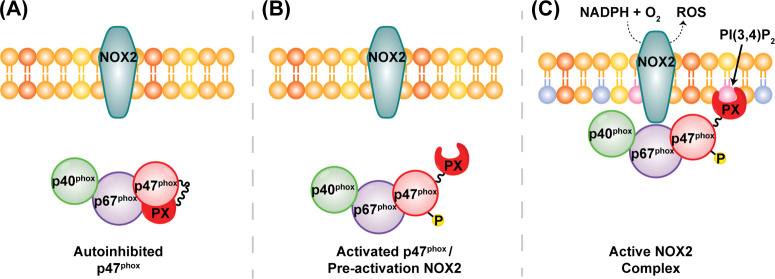
General scheme of NOX2 activation. (A) Autoinhibition of p47^phox^ PX domain constrains the activation complex of NOX2, consisting of p40^phox^, p67^phox^, and p47^phox^, to the cytosol resulting in deactivated NOX2. (B) Phosphorylation of p47^phox^ by PKC opens the p47^phox^ conformation and renders its PX domain accessible for lipid binding. Activation of p47^phox^ may also trigger trimerization of the activation complex. (C) Translocation of the activated trimeric complex is driven by p47^phox^ membrane anchoring through interactions between its PX domain and a PIP enriched phagosomal membrane, resulting in activation of NOX2 and production of ROS.

Activated NADPH oxidase 2 (NOX2) is mainly found in neutrophils and converts NADPH and O_2_ into superoxide radicals. The NOX2 membrane protein complex activates to create high concentrations of reactive oxygen species (ROS), which serve to eliminate microbes that have been engulfed into phagosomes, among other immunity-related functions.^1,2^ While important for innate immune responses, overactivation of NOX2 leads to a burst of ROS, which becomes cytotoxic and is implicated in diseases such as cardiovascular disease,^3−5^ cancers,^6,7^ and neurodegenerative diseases.^8−13^ The activated NOX2 complex consists of the membrane integral subunits gp91^phox^ and p22^phox^, as well as the cytosolic subunits p47^phox^, p40^phox^, p67^phox^, and the GTPase Rac.^14−16^ The p47^phox^ protein activates NOX2 through translocating an activation complex to the membrane. When NOX2 is dormant, p47^phox^ is autoinhibited and located within the cytosol [Figure 1A].^17,18^ This autoinhibited state is also generally believed to exist as a heterotrimer with p40^phox^ and p67^phox^^19,20^; however, other evidence suggests that the trimeric complex is formed only after p47^phox^ activation.^21^ To switch to the active state, protein kinase C must phosphorylate the C-terminal region of p47^phox^, rendering its N-terminal PX domain exposed and accessible for lipid recognition and binding [Figure 1B].^22,23^ Phosphoinositide binding by p47^phox^ translocates the entire trimeric activation complex, which also includes p40^phox^ and p67^phox^, from the cytosol to NOX2, which is located within the plasma membrane [Figure 1C]. Truncation of the PX domain from p47^phox^ or disruption of lipid binding through mutation is known to prevent activation of NOX2 by interrupting membrane translocation of p47^phox^ and the trimeric activation complex.^23−25^ As an essential mediator of NOX2 activity and regulator of immunity, a more complete understanding of the mechanism of action for p47^phox^ is critical.

The structure of the p47^phox^ PX domain (p47^phox^-PX) consists of a three-stranded antiparallel β-sheet followed by four α-helices and a polyproline II region.^26^ As compared to other PX domains, p47^phox^-PX is known to have relatively promiscuous binding for a variety of phosphoinositides, with phosphatidylinositol 3,4-bisphosphate [PI(3,4)P_2_] having the highest affinity.^27,28^ While no structure of p47^phox^-PX bound to lipids or mimics, such as phosphorylated inositols, are currently reported, the crystal structure of p47^phox^-PX displayed two bound sulfates within the α-helical and polyproline II surface.^22^ One sulfate is bound in the position corresponding to the typical PX domain phosphatidylinositol phosphate (PIP) binding site and the other in an atypical shallow pocket not previously implicated in PX domain PIP binding, indicating a possible secondary binding site. Subsequent studies elucidated the secondary, atypical, site as an anionic lipid binding site capable of binding to lipids such as phosphatidic acid and phosphatidylserine.^22^ These two sites may bind synergistically to different lipids within the plasma membrane; however, there is some evidence that the atypical site may be the primary PIP binding site.^22,24^

In stimulated neutrophils, the developing phagosomal membrane is enriched with 10-fold higher PI(3,4)P_2_ and 40-fold higher phosphatidylinositol 3,4,5-trisphosphate [PI(3,4,5)P_3_], as compared to the plasma membrane, which is composed of phosphatidylinositol 4,5-bisphosphate [PI(4,5)P_2_] as the highest abundance phosphoinositide.^29^ This enrichment is critical for the closure of the phagosomal cup and is depleted shortly after formation to develop a phosphatidylinositol 3-phosphate [PI(3)P] enriched membrane surface.^30,31^ Consistent with these findings, the PX domain of p47^phox^ has been proposed as the “main carrier” of the trimeric complex to the membrane in developing phagosomes. After formation of the active complex, anchoring through the p47^phox^-PX domain, and conversion of PI(3,4)P_2_ and PI(3,4,5)P_3_ to PI(3)P, the PX domain of p40^phox^ can aid in tethering the complex to the transmembrane NOX2.^32^

The highly phosphorylated inositol hexaphosphate (IP6) and inositol (1,3,4,5,6) pentakisphosphate (IP5) have been discovered to be the most abundant inositol phosphates (IPs) in stimulated neutrophils and are ubiquitous in mammalian cells, with IP6 and IP5 concentrations ranging from 5–15 μM or higher.^33,34^ Previous studies have shown a regulatory role of cytosolic IPs with PH-domain lipid-binding domains, which are similar to PX domains in function as PIP mediated membrane anchors. The Akt and PDK1 PH domains have been shown to be inhibited by highly phosphorylated IP variants IP5, IP6, and the pyrophosphate IP7.^35−37^ Another study has shown the membrane-binding C2 domains of granuphilin may also be regulated by cytosolic IPs.^38^ However, IPs can also regulate interactions through activation of PH domains as seen with ITK/BTK activation by IP4.^37^ Similarly, class I HDACs were shown to be activated by dissociating IP4(1,4,5,6) within the protein–protein interface.^39^ Inspired by these regulatory mechanisms, the relevant cytosolic concentrations of IPs, and their similarity to the phosphoinositide headgroup we sought to determine if a similar regulatory mechanism for p47^phox^-PX and NOX2 is possible.

In this study, we explore the binding of various IPs – IP6, IP5, inositol (1,3,4,5) tetrakisphosphate (IP4), and inositol (1,3,4) trisphosphate (IP3) – to the PX domain of p47^phox^. Through NMR observation, we determined binding of the IPs to the lipid binding site of the PX domain. Using an array of fluorescence assays, we discovered that IP6 is capable of inhibiting lipid binding and membrane anchoring of p47^phox^-PX with low micromolar potency. However, the other tested IPs were not capable of breaking such interactions in low- to midmicromolar concentrations. These results indicate that IP6 at physiologically relevant concentrations may serve as a regulator of NOX2 through inhibition of p47^phox^-PX membrane anchoring. Further, it suggests the possibility of using small molecules to inhibit NOX2 activity through the PX domain, which can be utilized for future therapeutic advances.

## Materials and Methods

### Protein Expression and Purification

The gene encoding human p47^phox^-PX (Residues 1–128 of p47^phox^, UniProt Accession ID P14598) with a TEV cleavable poly histidine tag was synthesized and subcloned into pET-28a by Genescript (Piscataway, NJ). The plasmid was transformed into BL21 (DE3) *E. coli* and grown on a LB-agar plate with kanamycin. Glycerol stocks were prepared from an overnight LB culture seeded with a single colony and were used to seed an overnight starter culture in M9 media at 37 °C. The overnight culture was pelleted and used to seed a 1L growth of M9 media containing 1 g/L ammonium chloride and 2 g/L d-glucose. Isotopic labeling for NMR experiments used 1 g/L ^15^N-ammonium chloride and 2 g/L ^13^C-d-glucose when necessary. All isotopes were purchased from Sigma-Aldrich (St. Louis, MO). Cells were grown to an OD_600_ of 1.0 at 37 °C/250 rpm before induction with 1 mM isopropyl β-d-1-thiogalactopyranoside and incubation overnight at 30 °C/200 rpm. The cells were harvested by an initial centrifugation at 4,000*g* for 20 min at 4 °C followed by a small volume resuspension of the pellet and an additional centrifugation at 10,000*g* for 20 min at 4 °C. The cell pellet was flash frozen and stored at −80 °C prior to lysis and purification. Cells were resuspended in lysis buffer [0.2 M Na_2_HPO_4_ (pH 7.4), 0.2 M NaCl, 0.5% (v/v) triton X-100, 1× protease inhibitor cocktail [Thermo Fisher Scientific (Waltham, MA)], 1 mM dithiothreitol (DTT), 5 mM MgCl_2_, 0.1 mg/mL lysozyme, and 6 μg/mL DNase [Bio Basic Inc. (Markham, ON)]] on ice for 2 h prior to sonication on ice. Cell lysate was centrifuged at 12,300*g* for 30 min at 4 °C and the pellet was discarded. His-tagged p47^phox^-PX was purified on ice over a Ni-NTA column using wash buffer [0.1 M Tris (pH 7.4), 0.1 M NaCl, 50 mM imidazole, 1 mM DTT] and elution buffer [0.1 M Tris (pH 7.4), 0.1 M NaCl, 250 mM imidazole, 1 mM DTT] prior to an overnight dialysis at 4 °C with an in-house expressed and purified TEV protease, MBP-TEVcs-His6-uTEV2Δ-R5^40^ [plasmid is a gift from Alice Ting, Addgene plasmid # 135456] to cleave the histidine tag. The Ni-NTA column purification process was repeated to fully purify p47^phox^-PX and a final dialysis at 4 °C into the desired assay buffer was performed. Protein concentration in buffer including DTT was calculated by a Bradford assay, and protein concentration in buffer with TCEP was calculated on a NanoDrop 2000 Spectrophotometer [Thermo Fisher Scientific (Waltham, MA)] using ε_280_ = 18,450 M^–1^ cm^–1^.

### NMR Spectroscopy

All NMR samples were prepared with either ^15^N- or ^15^N–^13^C-p47^phox^-PX in 50 mM Bis-Tris (pH 6.0), 100 mM NaCl, 1 mM DTT, 0.02% (v/v) sodium azide and 10% D_2_O as a lock solvent. Protein concentration was typically 100 μM in each sample, as confirmed by the Bradford Assay. NMR experiments were collected on a 600 MHz Bruker AVANCE III equipped with a TXI triple resonance probe at 25 °C. NMR assignments of the PX domain were transferred from a previous publication,^24^ then were confirmed, and expanded by collection of 3D HNCA, HN(CA)CB, and CBCA(CO)NH experiments on a 700 MHz Bruker AVANCE III at 25 °C.^41−43^ Standard Bruker pulse sequences were used with the HNCA collected with 64 scans and 1024 × 40 × 48 total points, the HN(CA)CB collected with 64 scans and 1024 × 40 × 64 total points, and the CBCA(CO)NH collected with 32 scans and 1024 × 40 × 96 total points. NMR data was processed with NMRPipe^44^ and analyzed using NMRFAM-Sparky.^45^

For NMR titrations, inositol phosphate stocks were prepared in the same NMR buffer, the IP6 stock was pH adjusted, and added in 0.25, 0.5, 2, and 4 to 1 mol equiv to the PX domain of p47^phox^. Due to the high buffering capacity of IPs, pH values were checked during titrations and corrected as needed. ^1^H–^15^N-HSQC spectra were collected at each titration point, using a standard Bruker pulse sequence, collected with 2048 × 100 total points and 16 or 32 scans. Chemical shift perturbations (CSPs) were calculated using weighted chemical shifts in the following formula^46^:
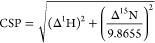
where Δ^1^H and Δ^15^N represent the changes in the ^1^H and ^15^N chemical shifts for each resonance. To calculate the binding affinity of IPs to the PX domain of p47^phox^, residues with ≥0.08 CSP at the highest titration point were fit to the following formula, which accounts for ligand depletion, on GraphPad Prism 10:
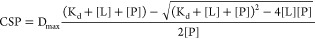
where CSP denotes the CSP of the individual residue at each IP concentration ([L]), D_max_ represents the CSP at saturation, [P] represents the concentration of p47^phox^-PX, and K_d_ represents the dissociation constant of p47^phox^-PX and IP. The formula was initially fit to each residue individually and any residue with R^2^ < 0.95 was removed before fitting the data globally to determine the overall binding affinity.

### Fluorescence Polarization

All fluorescence polarization (FP) experiments were performed using BODIPY-FL diC_6_–PI(3,4)P_2_ [Echelon Biosciences (Salt Lake City, UT)] as the reporting tracer. To determine the binding affinity of the fluorophore to p47^phox^-PX, a series of 2-fold dilutions, ranging from 10–0.02 μM protein, were performed in 50 mM Bis-Tris (pH 7.0), 100 mM NaCl, 1 mM MgCl_2_, and 1 mM DTT. Each sample contained 50 nM BODIPY-PI(3,4)P_2_ and was plated in triplicate on a black half area 96-well microplate [Corning (Corning, NY)]. The plate was centrifuged at 500 rpm for 30 s prior to analysis on a SpectraMax M5 [Molecular Devices (San Jose, CA)]. Measurements were taken using a monochromator with fixed excitation and emission wavelengths of 485 and 525 nm, respectively, and a cutoff of 515 nm. Raw data was converted to millipolarization (mP) using the SoftMax Pro software [Molecular Devices (San Jose, CA)]. mP values were then converted to anisotropy (A) using the following formula:
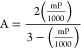


The binding affinity of BODIPY-PI(3,4)P_2_ to the PX domain was fit using GraphPad Prism 10 using the following equation, which accounts for ligand depletion through the titration experiment:
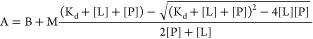
where A represents the anisotropy value at ligand concentration [L], B and M represent the baseline and max anisotropy respectively, [P] represents the protein concentration, and K_d_ represents the apparent dissociation constant between BODIPY-PI(3,4)P_2_ and p47^phox^-PX. One single outlier was removed for the measurement at 78 nM prior to fitting.

FP competition experiments were performed with inositol phosphate stocks in 50 mM Bis-Tris (pH 7.0), 100 mM NaCl, 1 mM MgCl_2_, and 1 mM DTT. A 2-fold dilution series of IP was performed, ranging from 50–0.05 μM IP in the presence of 2.5 μM p47^phox^-PX and 50 nM BODIPY-PI(3,4)P_2_. Samples were plated in triplicate on a black half area microplate, centrifuged at 500 rpm for 30 s, and incubated for 1 h prior to analysis. The data was converted to anisotropy and fit to a four-parameter, variable slope, dose–response equation on GraphPad Prism 10. The IC_50_ from the dose–response equation was converted to an inhibitory constant (K_i_) using a IC_50_-to-K_i_ calculator, which incorporated the following formula^47^:
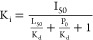
where I_50_ and L_50_ are the free concentrations of IP and BODIPY-PI(3,4)P_2_, respectively, at 50% inhibition, and P_0_ is the free concentration of p47^phox^-PX in the absence of IP.

### Liposome Preparation

To ensure proper liposome formation, phosphatidylinositol stocks were protonated prior to use adapted from a previously published protocol.^28^ In brief, lyophilized PI(3,4)P_2_ lipids were resuspended in CHCl_3_ and dried under nitrogen. Lipids were then further dried under a speed vacuum for 1 h followed by a resuspension into 2:1:0.01 CHCl_3_: MeOH: 1 M HCl. The suspension was allowed to sit at room temperature for 15 min before the drying process was repeated. Lipids were then reconstituted in 3:1 CHCl_3_: MeOH and dried as above. The process was repeated with CHCl_3_ before a final resuspension into CHCl_3_ at the desired concentration and storage of protonated stocks at −80 °C.

Lipids dissolved in chloroform were added to a vial at the desired 79:15:1:5 1-palmitoyl-2-oleoyl-glycero-3-phosphocholine (POPC): 1-palmitoyl-2-oleoyl-*sn*-glycero-3-phosphoethanolamine (POPE): diC_16_–PI(3,4)P_2_: Dansyl-PE [Avanti Lipids (Alabaster, AL)] ratio. The lipid suspension was mixed and dried under nitrogen with a continuous rotation in order to produce a thin lipid film. Dried lipids were further desiccated for 1.5 h using a speed vacuum. Drying was followed by a buffer resuspension into 5 mM Bis-Tris (pH 7.0), 100 mM NaCl, 1 mM MgCl_2_, and 0.5 mM TCEP. The lipid solution was sonicated using a microprobe in a NaCl-ice water bath for 5 min (0.6 s on/0.4 s off). The resulting liposome solution was briefly centrifuged in order to remove any potential debris. Liposomes were stored at 4 °C overnight before use and were used within 1 week.

### Dansyl-PE FRET Assay

The FRET assay was performed based on a previously published protocol,^48^ where intrinsic tryptophan fluorescence within the PX domain was utilized as the donor fluorophore and Dansyl-PE incorporated within liposomes as the acceptor fluorophore. Reactions were performed with 2.5 μM p47^phox^-PX and 125 μM total accessible lipid (i.e., exposed on the outer surface of the liposome), which is approximated as one-half the total lipid, in 5 mM Bis-Tris (pH 7.0), 100 mM NaCl, 1 mM MgCl_2_, and 0.5 mM TCEP. Samples were mixed for 40 s after each IP6 addition and were analyzed on an OLIS 14 TB NIR/CD Spectrophotometer [Olis (Bogart, GA)] equipped with a UV/vis photon counter 230–870 nm fluorescence detector. Fixed excitation and emission wavelengths of 284 and 520 nm were used with a 1 nm excitation slit width and a 12.5 nm emission slit width. To correct for a Wood’s anomaly at 500–530 nm, a horizontal calcite polarizer was placed on the excitation path and a vertical MgFl polarizer on the emission path.^49^ Each titration point measurement was integrated over 10 s and measured in triplicate. Titrations were repeated in triplicate and corrected for dilution or IP6 effects by completing triplicate “blank” titrations of IP6 in the presence of only liposomes, which were averaged for the correction. The corrected data was normalized and fit to the following formula on GraphPad Prism 10 to calculate the IC_50_:
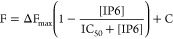
where Δ*F*_max_ is the maximum FRET signal of p47^phox^-PX and Dansyl-PE incorporated into liposomes before addition of IP6. The data was normalized so that Δ*F*_max_ = 1 and C = 0.

### Docking

Molecular docking of IP6 to the PX domain of p47^phox^ was performed using AutoDock Vina 1.1.2 and AutoDock Tools.^50^ The 3D structural coordinates of IP6, at the correct protonation state for pH = 7.0, was generated in MarvinSketch 21.3.0 [ChemAxon (Budapest, Hungary)].^51^ Hydrogens were added to the NMR solution structure of p47^phox^-PX (PDB: 1GD5)^26^ using AutoDock Tools. A docking grid encompassing the surface of PX domain residues that displayed shifting in the NMR titration of IP6 was used. Simulations were run with an exhaustiveness setting of 400. Docking results were analyzed using UCSF Chimera 1.16.

## Results and Discussion

### IPs Bind to the p47^phox^-PX Domain

**Figure 2:**
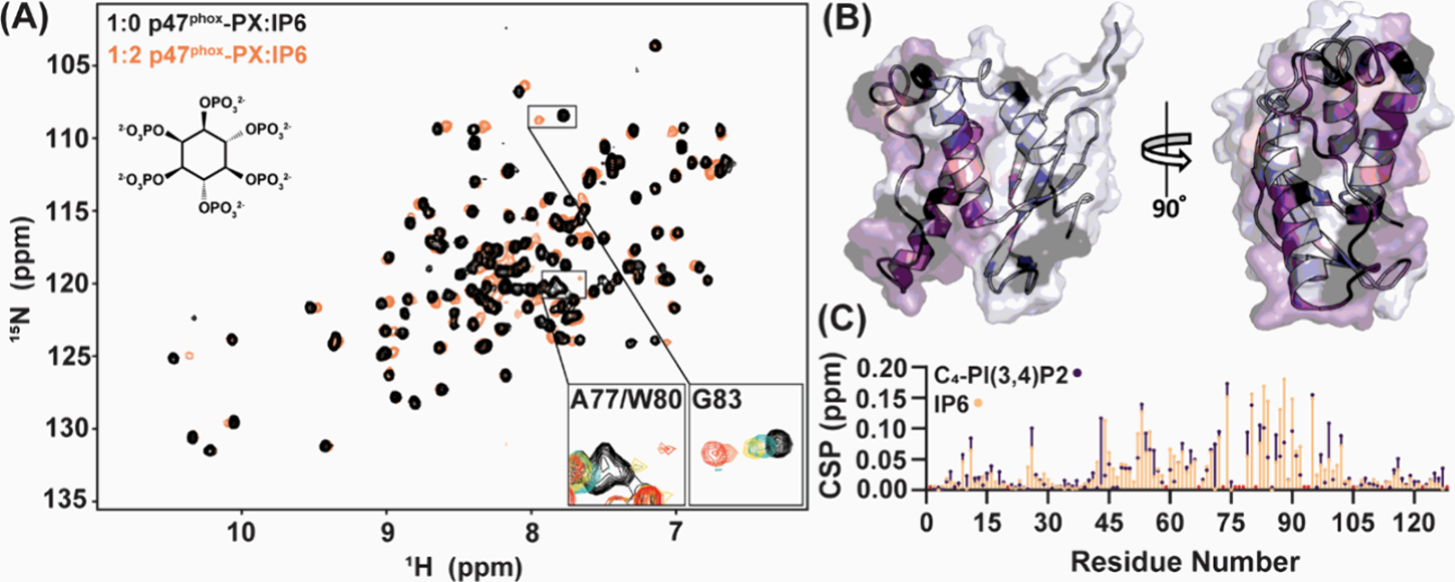
IP6 binds to the PX domain of p47^phox^ as observed by NMR. (A) ^1^H–^15^N-HSQC overlay of 1:0 and 1:2 molar ratios of p47^phox^-PX:IP6. Insets depict a titration series including 1:0.25, 1:0.5, 1:2, and 1:4 molar ratios of PX domain:IP6. High-affinity binding is implicated through line-broadening of resonances. (B) Mapping of CSPs upon addition of 2× IP6 to the PX domain (PDB: 1GD5). Residues depicting 1 and 2 standard deviations of a 20% trimmed mean are in pink and purple, respectively. Residues with unobservable or line-broadened peaks are depicted in black. (C) Comparison of CSPs upon 2× C_4_–PI(3,4)P_2_ (purple) or IP6 (orange). Residues with unobservable or line-broadened peaks are shown as red circles offset from the *x*-axis.

To understand and characterize potential PX domain interactions with IPs, we used protein-observed ^1^H–^15^N-HSQC NMR experiments to observe binding interactions. Significant chemical shift perturbations (CSPs) and resonance line-broadening were observed beginning with the addition of 0.25:1 molar ratios of IP6 to protein [Figure 2A; Figure S1A]. Resonances corresponding to residues A77, W80, and F81 had the most severe line-broadening effects, nearly completely disappearing at this molar ratio. This is consistent with ligand exchange in the intermediate NMR time scale, which is often observed for ligand binding in the low micromolar, or lower, range.^52^ At higher ligand ratios, multiple residues appeared to reach saturation. To determine the approximate binding surface of IP6, the CSPs of 2X IP6 were mapped to the previously published NMR structure [Figure 2B; Figure S1B].^26^ Residues with CSPs of 1 or 2 standard deviations from a 20% trimmed mean encompassed the majority of residues in the known p47^phox^-PX lipid binding site. To understand if the binding of IP6 is similar to binding to PIP substrates, we compared these CSPs to those induced with the addition of C_4_–PI(3,4)P_2_, a water-soluble version of the PX domain’s highest affinity lipid [Figure S2]. We observed that the amino acid specific shifting, and the magnitude of CSPs, induced by IP6 was very similar to that of C_4_–PI(3,4)P_2_, indicating a similar binding mode of IP6 within the lipid headgroup recognition site [Figure 2C]. However, due to the line-broadening observed, NMR titration would not be suitable for affinity determination of IP6.^52^

**Figure 3:**
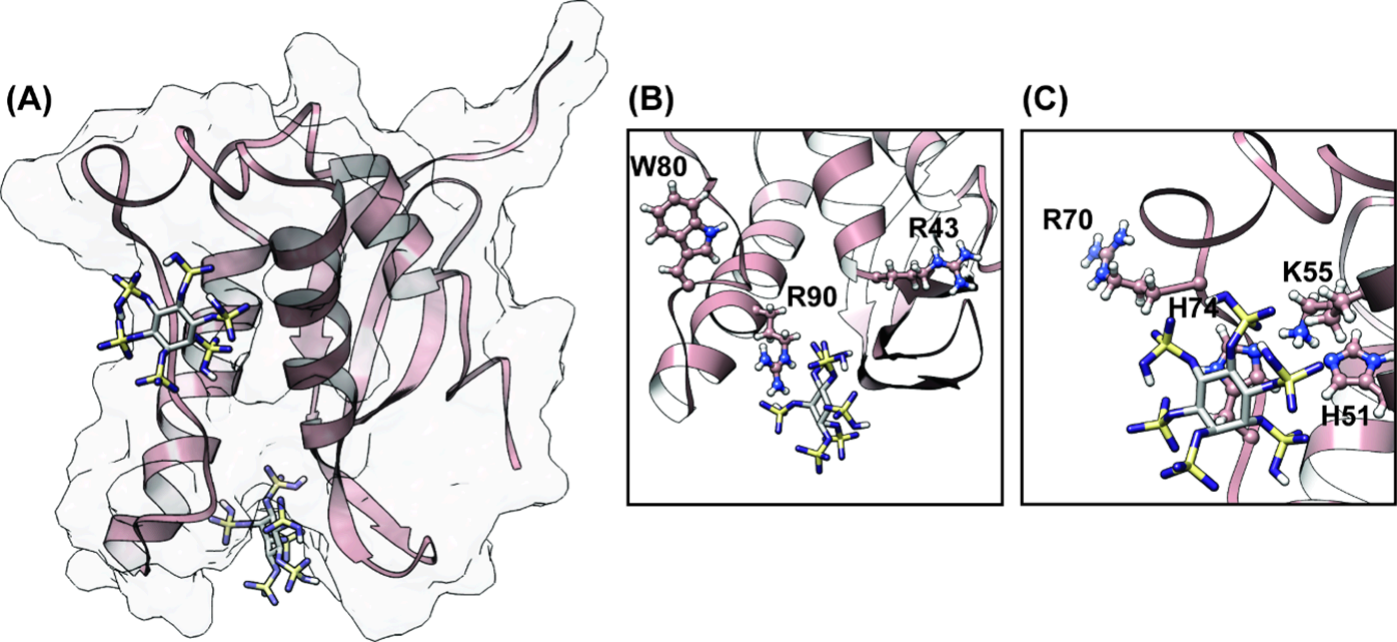
Docking simulation of IP6 against the PX domain of p47^phox^ (PDB: 1GD5). (A) Two highest scoring docking poses show IP6 bound either within the typical PIP binding pocket or the atypical shallow anionic lipid binding pocket. (B) IP6 bound within the PIP binding site is flanked by three residues known to be important to lipid binding (R43, W80, R90). (C) IP6 bound within the atypical binding site is flanked by four residues critical for lipid recognition (H51, K55, H74, and R70).

Molecular docking was used to visualize potential interactions between IP6 and p47^phox^-PX. The residues that had corresponding CSPs greater than 1 standard deviation against the trimmed mean were assumed to be in or proximal to the IP6 binding site. The docking simulations were constrained to a box that included only these and neighboring residues within the PX domain NMR structure (PDB: 1GD5).^26^ The results displayed various binding poses within the typical PIP binding site, and one within the atypical anionic lipid binding site of the PX domain [Figure 3A].^22^ The highest ranked binding position is within the typical PIP binding site surrounded by residues flanking the sulfate in the crystal structure, specifically, including residues R43, W80, and R90 [Figure 3B]. Through mutational analysis and SPR, these residues were shown to be important for PI(3,4)P_2_ binding and reduced the affinity by 29–245 fold as compared to the unmutated PX domain.^22^ The seventh highest docking position displayed IP6 bound within the shallow atypical anionic lipid binding site [Figure 3C]. Similarly, residues H51, K55, R70, and H74 flank this binding pose and have been shown to reduce binding to lipids through NMR titrations with C_4_–PI(3,4)P_2_.^24^ Importantly, residue K55 is in close proximity to the docked IP6 and is known to abrogate PIP binding and drastically reduces NOX2 activation.^24^ These results in combination with our NMR titration study indicate that IP6 binds within the typical PIP binding site of p47^phox^-PX and may also interact with the atypical secondary anionic binding site, which has also been implicated as possibly being the primary PI(3,4)P_2_ binding site.^24^ However, while docking shows two potential binding sites, NMR study of the interaction between IP6 and p47^phox^-PX does not distinguish between specific binding modes. We further pursued competition studies to ascertain IP6’s ability to break the lipid interactions of p47^phox^-PX, despite where it may bind within the domain.

**Figure 4:**
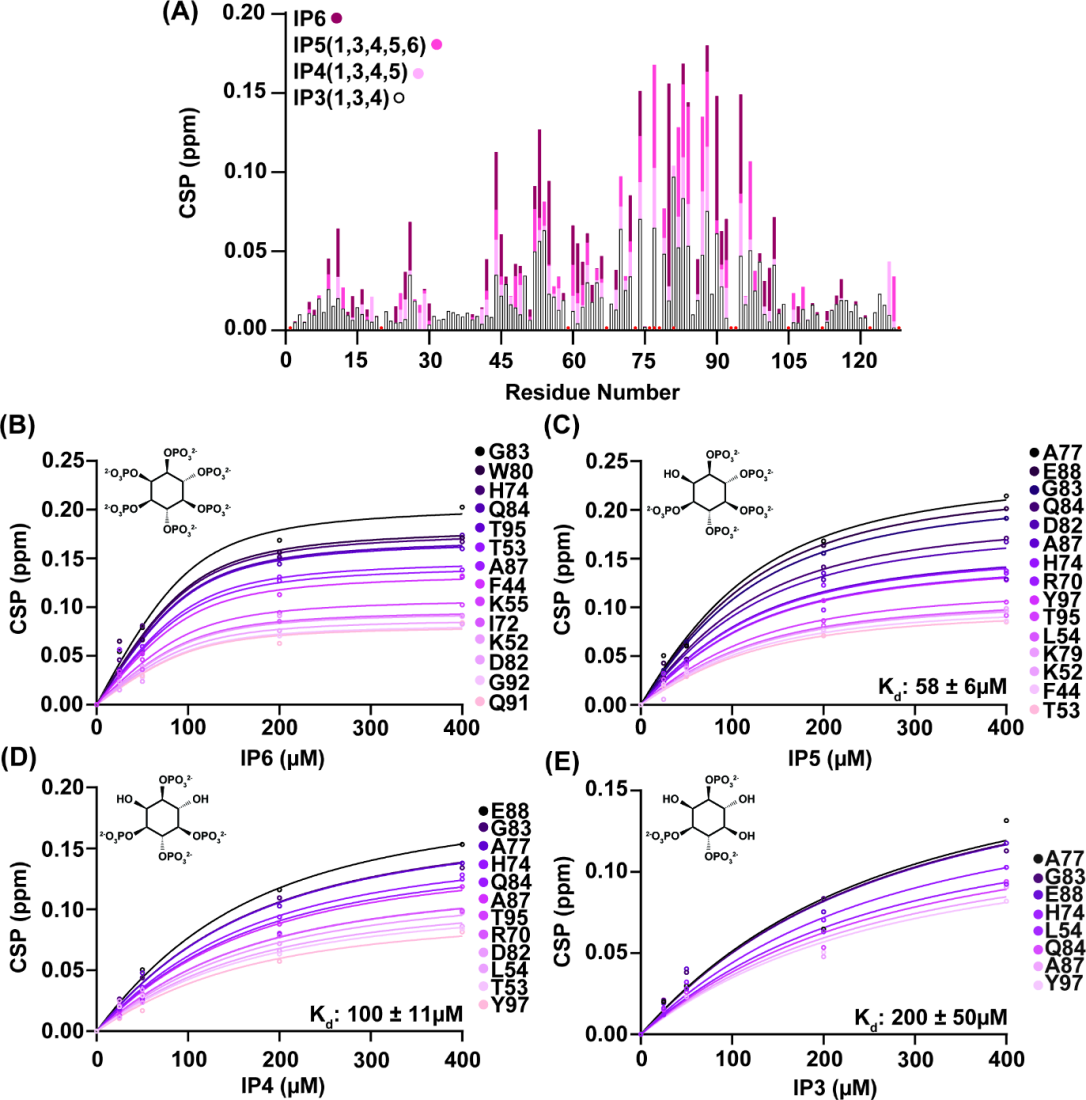
Apparent binding affinities of tested IPs as observed by NMR titration. (A) CSPs per residue of p47^phox^-PX in the presence of IP6, IP5, IP4, and IP3. Comparison of CSPs induced by IPs display larger shifting and tighter binding to the PX domain with higher degrees of phosphorylation around the inositol ring. Residues that line-broadened and/or were unobservable are denoted as red circles offset from the *x*-axis. (B) Titration of the PX domain with IP6. Line-broadening prevented accurate *K*_d_ fitting; curves are included here only as a guide for the eye. NMR titrations of the PX domain with IP5(1,3,4,5,6) (C), IP4(1,3,4,5) (D), and IP3(1,3,4) (E) were used to calculate K_d_ values. Curves in panels (C)–(E) represent the fit of each residue to a global K_d_ value, where all resonances with a CSP > 0.08 and R^2^ > 0.95 when fit individually were included.

NMR titrations were repeated for other IPs – IP5(1,3,4,5,6), IP4(1,3,4,5), and IP3(1,3,4) – to further understand IP binding to the PX domain. We selected IP5(1,3,4,5,6) and IP4(1,3,4,5) since they are major isoforms observed in mammalian cells and neutrophils, respectively,^33,34^ while IP3(1,3,4) was selected due to its similarity to the known PI(3,4)P_2_ substrate headgroup. Each titration resulted in high-quality spectra with well-defined CSPs of 1 and 2 standard deviations located in the binding site of p47^phox^-PX [Figures S3–S5]. Mapping the chemical shift perturbations reveals that the binding sites for all IPs are similar, with a trend of decreasing concentration-dependent CSPs as phosphorylation around the ring decreases is apparent [Figure 4A]. Strikingly, while IP3(1,3,4) most closely imitates the headgroup of C_4_–PI(3,4)P_2,_ the CSPs of p47^phox^-PX upon addition of IP3 are reduced as compared to C_4_–PI(3,4)P_2_ addition. Since IP3 is essentially the headgroup of C_4_–PI(3,4)P_2_, these results demonstrate the importance of either the glycerol backbone or acyl-tail in enhancing the affinity of the lipid to the PX domain. This suggests that binding of p47^phox^-PX to its highest affinity PIP, PI(3,4)P_2_, is not solely driven by headgroup interactions. While intermediate-exchange induced line-broadening precluded use of NMR for IP6 affinity determination, the presence of this phenomenon suggests an approximately low-μM or lower affinity.^52^ Line-broadening was not observed in the titrations of IP5, IP4, or IP3, allowing for affinity measurements by NMR. Residues with a CSP equal to, or above, 0.08 at the highest titration point were selected and globally fit to common K_d_ values for each IP titration [Figure 4B–E]. IP5 fit to an affinity of 58 ± 6 μM, IP4 fit to an affinity of 100 ± 11 μM, and IP3 fit to an affinity of 200 ± 50 μM. Additionally, inositol-1-phosphate was titrated up to 2.4 mM and showed a much lower binding affinity, with a K_d_ of 870 ± 120 μM [Figure S6]. Inositol, which lacks phosphates entirely, was also tested however no significant CSPs were observed up to 64 mM [Figure S7]. These results indicate that higher degrees of inositol phosphorylation resulted in greater binding affinities and a lower K_d_.

### Evaluating IP6 as a Competitive Inhibitor of PI(3,4)P_2_ Binding to p47^phox^-PX

**Figure 5:**
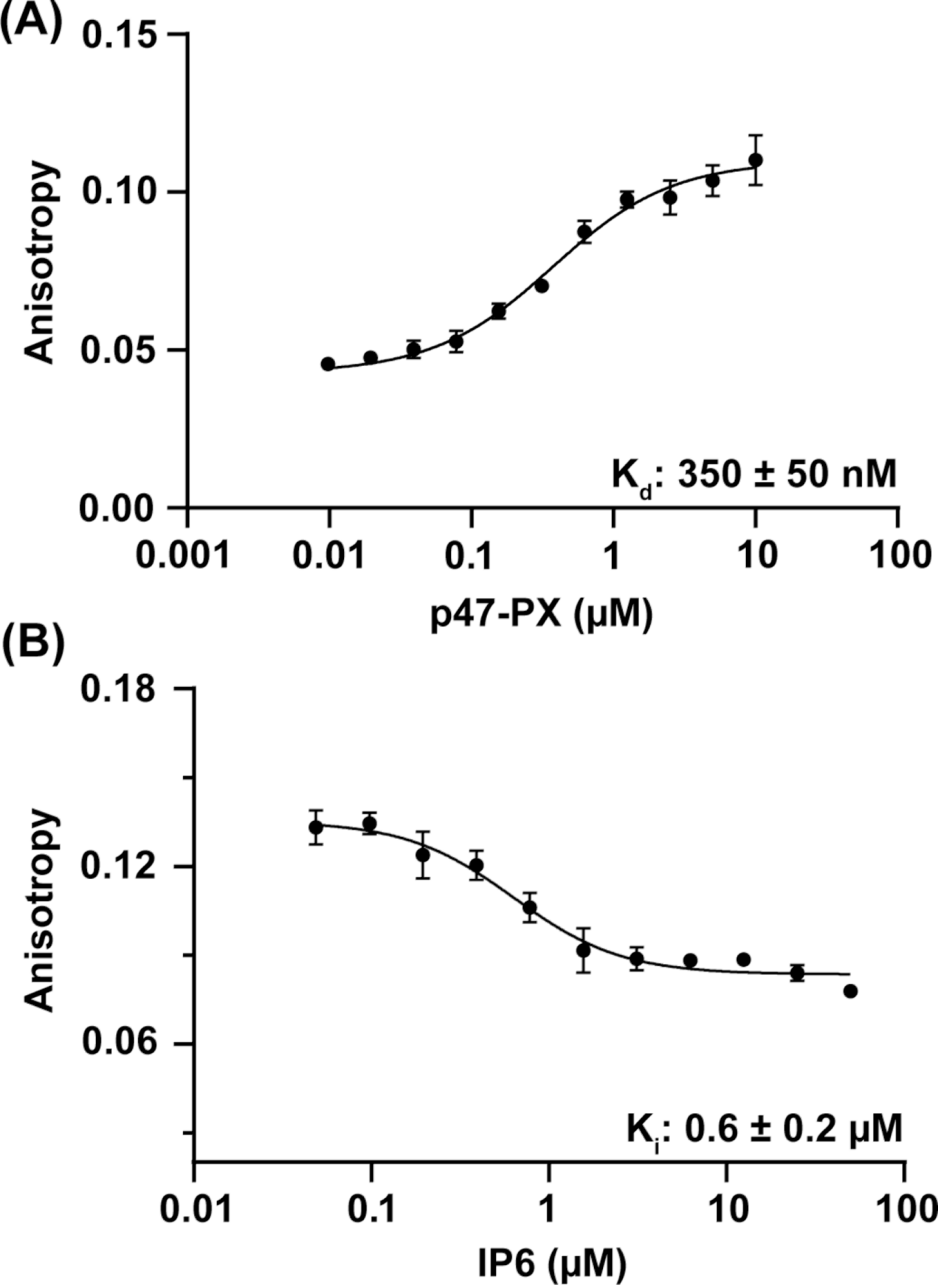
Fluorescence polarization study to assess IP6 as a competitive inhibitor. (A) Binding of tracer BODIPY-PI(3,4)P_2_ to p47^phox^-PX and associated fit for K_d_ determination. (B) Displacement of BODIPY-PI(3,4)P_2_ tracer from the PX domain upon addition of IP6 and associated fit. Error bars represent standard deviation of experimental replicates; those not visible are smaller than the data points.

The NMR result revealed that IP6, which is an abundant cellular component, has the highest affinity toward p47^phox^-PX of the tested IPs, though the exact affinity for IP6 could not be measured due to line-broadening. Since NMR is unsuitable for affinity determination of IP6, we sought an alternate method. Additionally, the NMR and docking results did not unambiguously resolve whether IP6 binds to the same site as PI(3,4)P_2_. To understand if IP6 may act as a competitive inhibitor of PI(3,4)P_2_, we employed a fluorescence polarization (FP) competition assay using the fluorescently labeled tracer, BODIPY FL PI(3,4)P_2_. Not only does this method reveal whether IP6 binding is competitive with lipid binding, but it will provide an affinity for IP6 in the form of an inhibition constant (K_i_). An initial binding measurement between the tracer and p47^phox^-PX was performed in order to determine its binding affinity to the PX domain. We determined that the K_d_ of BODIPY-PI(3,4)P_2_ was 350 ± 50 nM [Figure 5A]. We note that our experiments were performed in concentrations well under the expected CMC of short-chain PIPs, which is expected to fall in the millimolar range.^53^

IP6 was analyzed against a constant concentration of BODIPY-PI(3,4)P_2_ (50 nM) and p47^phox^-PX (2.5 μM) in order to determine its ability to break the interaction. Concentration of Mg^2+^ and pH are important contributors to the ionization of IPs and physiologically relevant conditions for each were tested.^54,55^ Since IP6 is a known chelator, in order to minimize artifacts or false positives, we performed FP and all following experiments under physiological MgCl_2_ concentrations.^54,56^ We chose to perform these measurements at the cytosolic pH of neutrophils, which is known to be around 7.^55^ Results revealed that IP6 is capable of breaking the lipid binding event with very low micromolar potency, with a K_i_ of 0.6 ± 0.2 μM [Figure 5B]. Neutrophil cytosolic pH is known to drop upon phagocytosis; however, we found only a minor effect on inhibition at a lower pH [Figure S8A]. We also found that the absence of MgCl_2_ has little to no effect on the p47^phox^-PX and IP6 interactions [Figure S8B]. As the next highest affinity IP, we tested IP5 to see if there was any capacity to break the BODIPY-PI(3,4)P_2_ and PX domain interactions; however, there was no significant observed competition at the concentrations tested [Figure S8C]. This data further supports potential negative regulation of p47^phox^-PX by IP6, which is ubiquitously abundant in cells at concentrations well above the K_i_ determined here.

### Membrane Anchoring of p47^phox^-PX Is Inhibited by IP6

While we found that multiple different IPs can bind to the PX domain and IP6 is capable of inhibiting the single lipid binding interactions of p47^phox^-PX, further study was needed to ascertain the ability of IP6 to break the membrane interaction and anchoring event. This evidence is important in the context of regulation since membrane localization is the primary function of the PX domain and a major driver of NOX2 activation. To investigate, we performed a FRET-based assay using intrinsic tryptophan as the donor fluorophore and Dansyl-PE incorporated into liposomes as the accepting fluorophore.^57^ As a tryptophan-containing protein anchors to a Dansyl-labeled liposome, the acceptor Dansyl fluorescence is expected to increase. Previously, this assay was used to characterize IP6 inhibition of a membrane binding event for C2 domains.^38,48^ In addition to the acceptor fluorophore lipid, the liposomes contained PI(3,4)P_2_, which is the PIP with the highest affinity for p47^phox^-PX,^27^ and a mixture of POPC and POPE at overall molar ratios of 79:15:1:5 POPC:POPE:diC_16_–PI(3,4)P_2_:Dansyl-PE. A physiologically relevant MgCl_2_ concentration of 1 mM and a pH of 7.0 were used.^54−56^ This assay would reveal whether IP6 is able to inhibit the interaction of p47^phox^-PX and PIP within the context of a membrane, thus preventing membrane anchoring.

**Figure 6:**
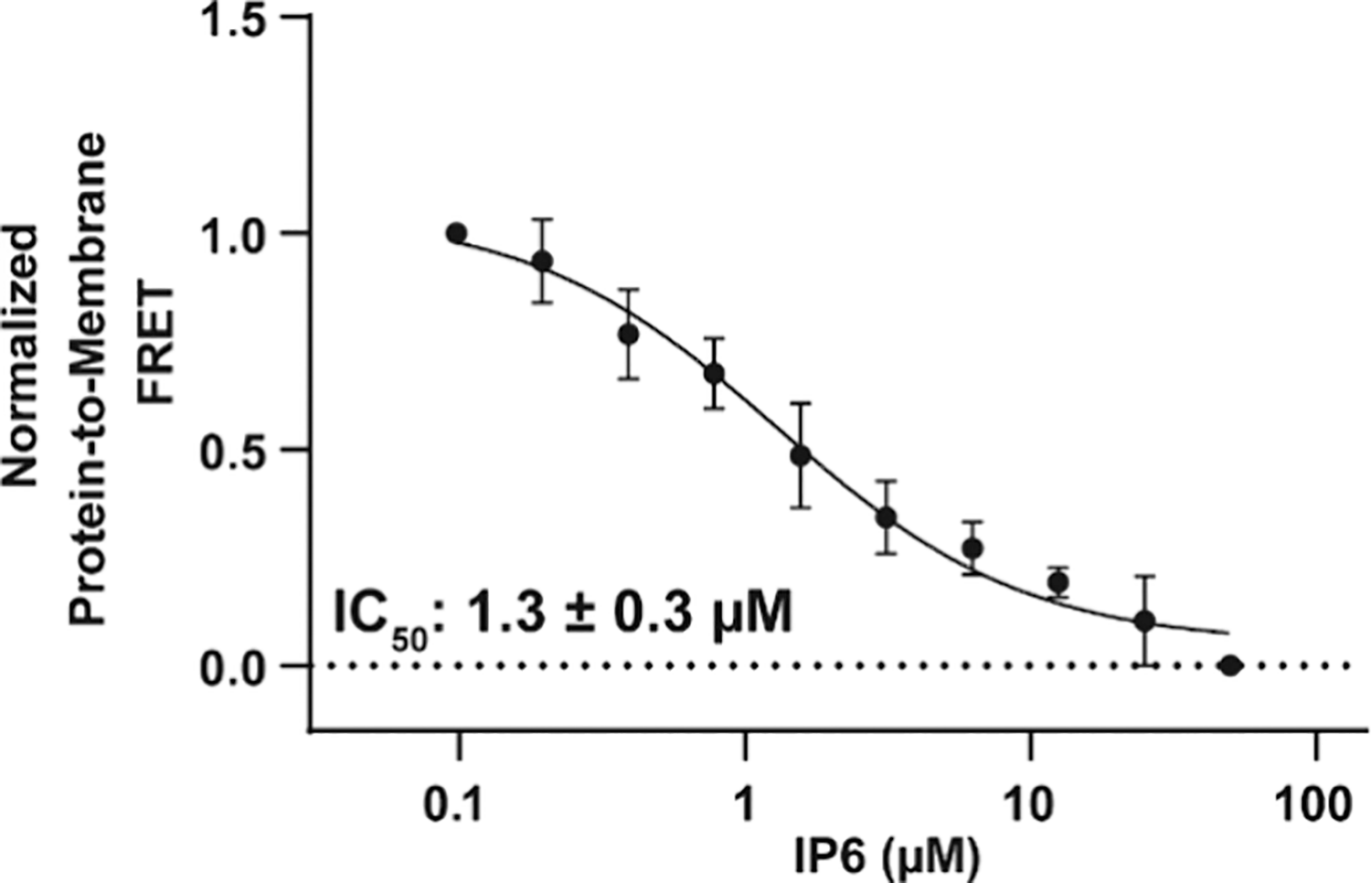
IP6 acts as an inhibitor of membrane anchoring of p47^phox^-PX with low micromolar potency as seen by a liposome FRET assay. Donor fluorophore is the PX domain intrinsic tryptophans, and acceptor fluorophore is Dansyl-PE incorporated into liposomes. Titrations of IP6 against p47^phox^-PX and liposomes were completed in triplicate and normalized. Error bars represent the deviation of individual normalized titrations.

An increase in Dansyl-PE fluorescence signal was observed upon p47^phox^-PX addition to the liposome solution corresponding to the localization and binding of p47^phox^-PX to the liposome. A decrease in signal is expected to be observed as the PX domain is inhibited from binding to the membrane.^38,48^ Triplicate titrations of IP6 against p47^phox^-PX and Dansyl-PE incorporated liposomes were performed and revealed inhibition of membrane anchoring with an apparent IC_50_ of 1.3 ± 0.3 μM [Figure 6]. While the IC_50_ of this assay is slightly higher, it is consistent with the inhibitory constant of IP6 as observed by the FP competition assay (K_i_ 0.6 ± 0.2 μM) and relates to breaking of PX domain-membrane interactions as compared to a single lipid, BODIPY-PI(3,4)P_2_. This demonstrates that not only does IP6 act as a competitive inhibitor for a water-solubilized analog of PI(3,4)P_2_, IP6 also acts as an inhibitor to membrane anchoring at approximately 1 μM. These results reveal that inhibition occurs at concentrations below the IP6 concentration found ubiquitously within cells, indicating a possible regulatory mechanism.

## Conclusions

In this study, we discovered a potential novel regulatory mechanism of p47^phox^-PX membrane interactions and NOX2 activation. Through an array of NMR, fluorescence polarization, and FRET assays, we determined that IP6 is capable of breaking the lipid and membrane interactions of the PX domain with low micromolar potency. IP6 is found ubiquitously in cells at 5–15 μM, above the K_i_ and IC_50_ found here, which indicates that IP6 may act as a regulator of p47^phox^-PX membrane translocation. Regulation by IPs may prevent aberrant attachment of p47^phox^ to membranes since PIPs are signaling molecules in a variety of cascades in the plasma and other membranes. Many cytosolic facing membranes and the inner leaflet of the plasma membrane in particular are known to be enriched in PIP lipids compared to most other cellular membranes.^58^ With the known promiscuity of p47^phox^-PX toward a variety of PIPs, it is unknown how p47^phox^ is able to selectively translocate to the phagosomal membrane while avoiding anchoring to other PIP containing membranes. Inhibition by IP6 may prevent anchoring of activated p47^phox^ to nonphagosomal, PIP enriched membranes. Neutrophil activation results in a high degree of enrichment of PIPs in phagosomes, particularly PI(3,4)P_2_, PI(3,4,5)P_3_, and PI(3)P, which may overcome the ability of IP6 to inhibit the membrane localization allowing p47^phox^ to activate NOX2. Additionally, concentrations of IPs are known to be in flux upon neutrophil activation, which may help modulate the inhibitory effect and therefore NOX2 activation.^33^ While IP6 may have a role in regulating the activation complex, this study was conducted without other activation complex partners and so further investigation would be needed to understand if IP6 affects interactions between p47^phox^, p67^phox^, and p40^phox^. IPs have been found to be important in the regulation of other PIP recognition proteins with PH domains, such as Akt, PDK1, and ITK/BTK, and the C2 domains of granuphilin.^35−38^ The results here highlight an emerging paradigm of IP regulation of PH and PX domain recognition of PIPs in membranes. Not only does this study suggest a cellular regulatory mechanism but also indicates that small-molecule inhibition of the PX domain may be possible as an alternative approach to inhibition of NOX2 activity in disease states such as cardiovascular diseases, cancers, and neurodegenerative diseases.

## Supporting Information Available

NMR titration of IP6; NMR titration of C_4_–PI(3,4)P_2_; NMR titration of IP5(1,3,4,5,6); NMR titration of IP4(1,3,4,5); NMR titration of IP3(1,3,4); NMR titration of inositol-1-phosphate (I1P); NMR spectral overlay of p47^phox^-PX with and without inositol; fluorescence polarization competition measurements of IP5(1,3,4,5,6) and IP6 in low-pH or no Mg^2+^ conditions (PDF)
