# Supplementary material for: Inositol Hexaphosphate as an Inhibitor and Potential Regulator of p47phox Membrane Anchoring
Source: Biochemistry. 2024 Apr 26;63(9):1097–106. doi: 10.1021/acs.biochem.4c00117 (PMC11080064; doi:10.1021/acs.biochem.4c00117)
Supplement: Supplementary file 1 — bi4c00117_si_001.pdf [file bi4c00117_si_001.pdf]

Supporting Information for: Inositol Hexaphosphate as an Inhibitor and Potential Regulator of p47^{phox} Membrane Anchoring

Angela M. Develin[1], *Brian Fuglestad*[*1,2]

[1] Department of Chemistry, Virginia Commonwealth University, Richmond, Virginia 22384, United States.

[2] Institute for Structural Biology, Drug Discovery and Development, Virginia Commonwealth University, Richmond, Virginia 23219, United States.

[*]Correspondence should be addressed to: fuglestadb@vcu.edu

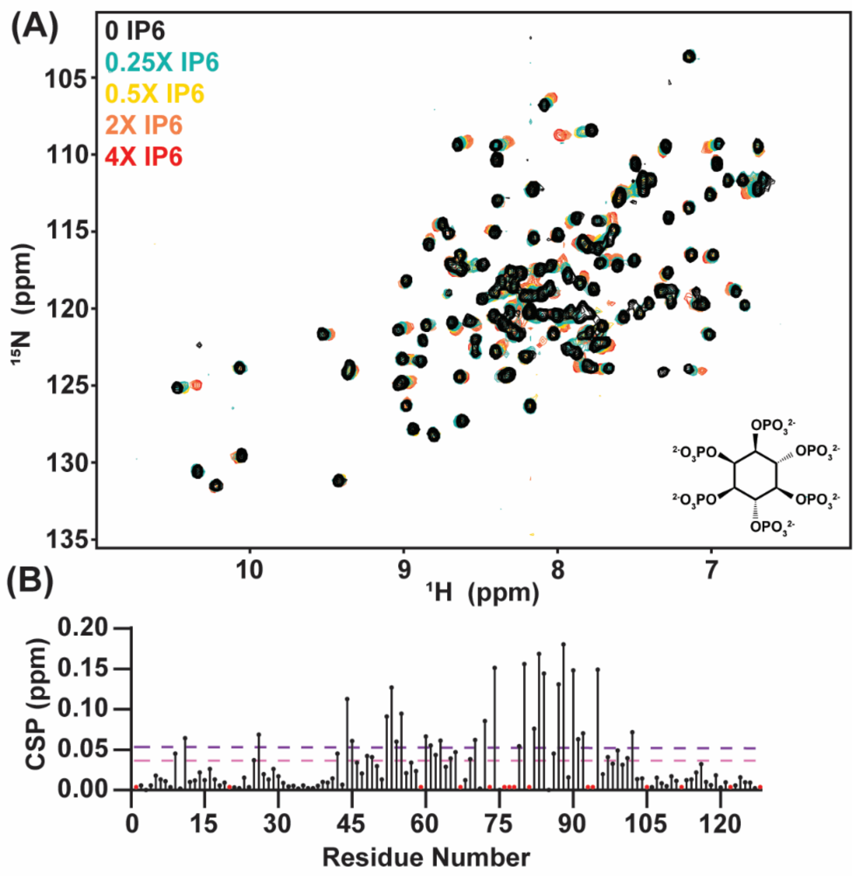

Figure S1. NMR titration of IP6. (A) Spectral overlay of titration encompassing 0 – 4:1 IP6:p47^{phox}-PX with 100 μM PX domain. (B) Chemical shift perturbations per residue of p47^{phox}-PX upon addition of 2X IP6. Pink and purple dashed lines represent 1 and 2 σ of a 20% trimmed mean, respectively. Line-broadened or unobservable residues are depicted as red circles offset from the x-axis.

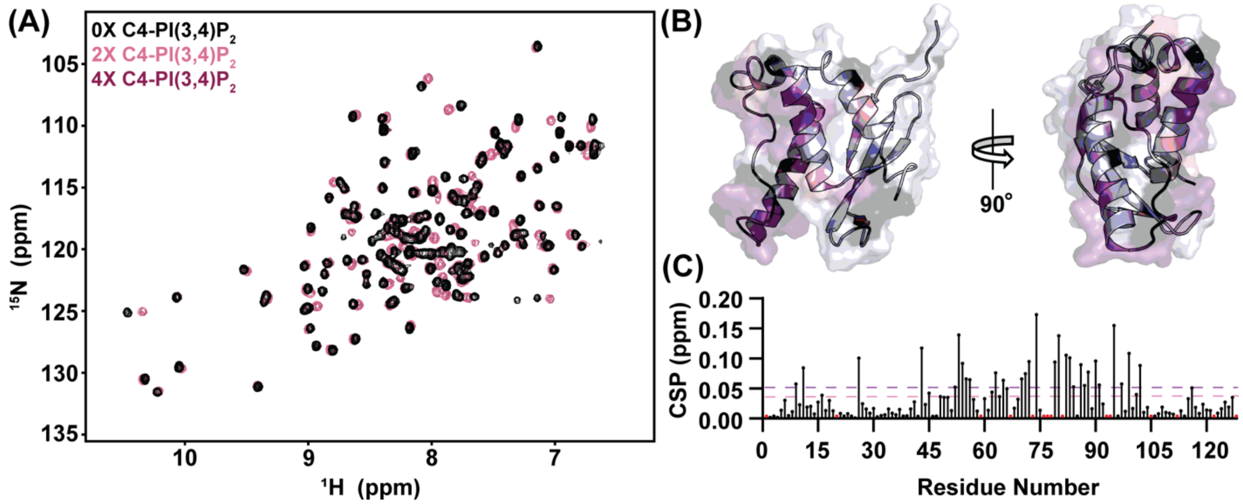

Figure S2. NMR titration of C_4-PI(3,4)P_2. (A) Spectral overlay of titration encompassing 0 – 4:1 PI(3,4)P_2:p47^{phox}-PX with 100 μM PX domain. (B) 2X C_4-PI(3,4)P_2 CSPs mapped on to the PX domain (PDB: 1GD5). Residues highlighted in pink and purple represent CSPs greater than 1 and 2 σ of a 20% trimmed mean, respectively. Line-broadened or unobservable residues are highlighted in black. (C) Chemical shift perturbations per residue of p47^{phox}-PX upon addition of 2X C_4-PI(3,4)P_2. Pink and purple dashed lines represent 1 and 2 σ of a 20% trimmed mean, respectively. Line-broadened or unobservable residues are depicted as red circles offset from the x-axis.

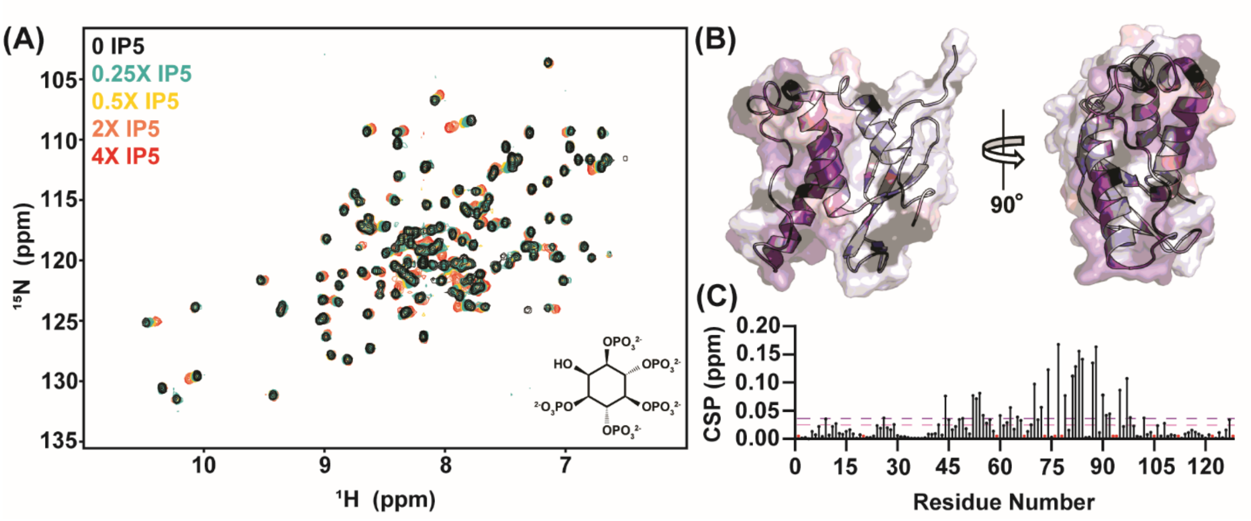

Figure S3. NMR titration of IP5(1,3,4,5,6). (A) Spectral overlay of titration encompassing 0 – 4:1 IP5:p47^{phox}-PX with 100 μM PX domain. (B) 2X IP5 CSPs mapped on to the PX domain (PDB: 1GD5). Residues highlighted in pink and purple represent CSPs greater than 1 and 2 σ of a 20% trimmed mean, respectively. Unobservable residues are highlighted in black. (C) Chemical shift perturbations per residue of p47^{phox}-PX upon addition of 2X IP5. Pink and purple dashed lines represent 1 and 2 σ of a 20% trimmed mean, respectively. Unobservable residues are depicted as red circles offset from the x-axis.

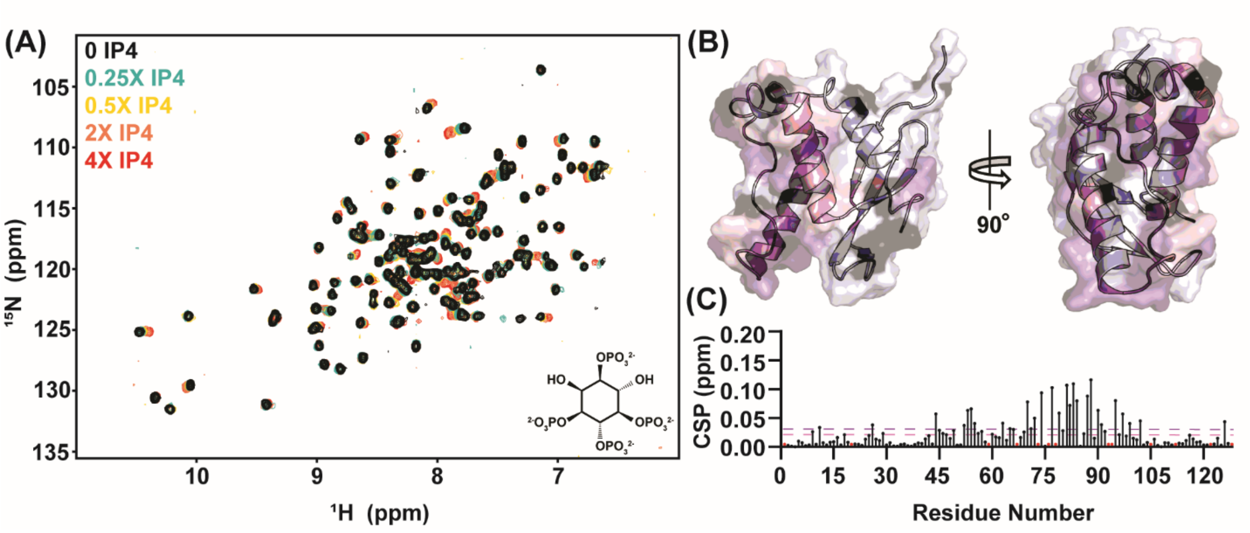

Figure S4. NMR titration of IP4(1,3,4,5). (A) Spectral overlay of titration encompassing 0 – 4:1 IP4:p47^{phox}-PX with 100 µM PX domain. (B) 2X IP4 CSPs mapped on to the PX domain (PDB: 1GD5). Residues highlighted in pink and purple represent CSPs greater than 1 and 2 σ of a 20% trimmed mean, respectively. Unobservable residues are highlighted in black. (C) Chemical shift perturbations per residue of p47^{phox}-PX upon addition of 2X IP4. Pink and purple dashed lines represent 1 and 2 σ of a 20% trimmed mean, respectively. Unobservable residues are depicted as red circles offset from the x-axis.

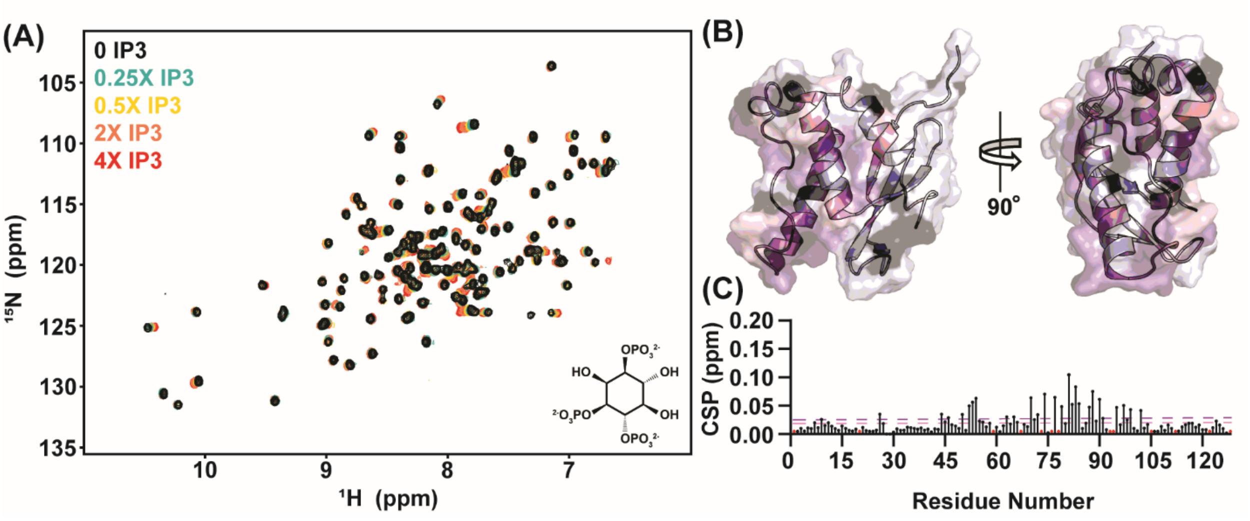

Figure S5. NMR titration of IP3(1,3,4). (A) Spectral overlay of titration encompassing 0 – 4:1 IP3:p47^{phox}-PX with 100 μM PX domain. (B) 2X IP3 CSPs mapped on to the PX domain (PDB: 1GD5). Residues highlighted in pink and purple represent CSPs greater than 1 and 2 σ of a 20% trimmed mean, respectively. Unobservable residues are highlighted in black. (C) Chemical shift perturbations per residue of p47^{phox}-PX upon addition of 2X IP3. Pink and purple dashed lines represent 1 and 2 σ of a 20% trimmed mean, respectively. Unobservable residues are depicted as red circles offset from the x-axis.

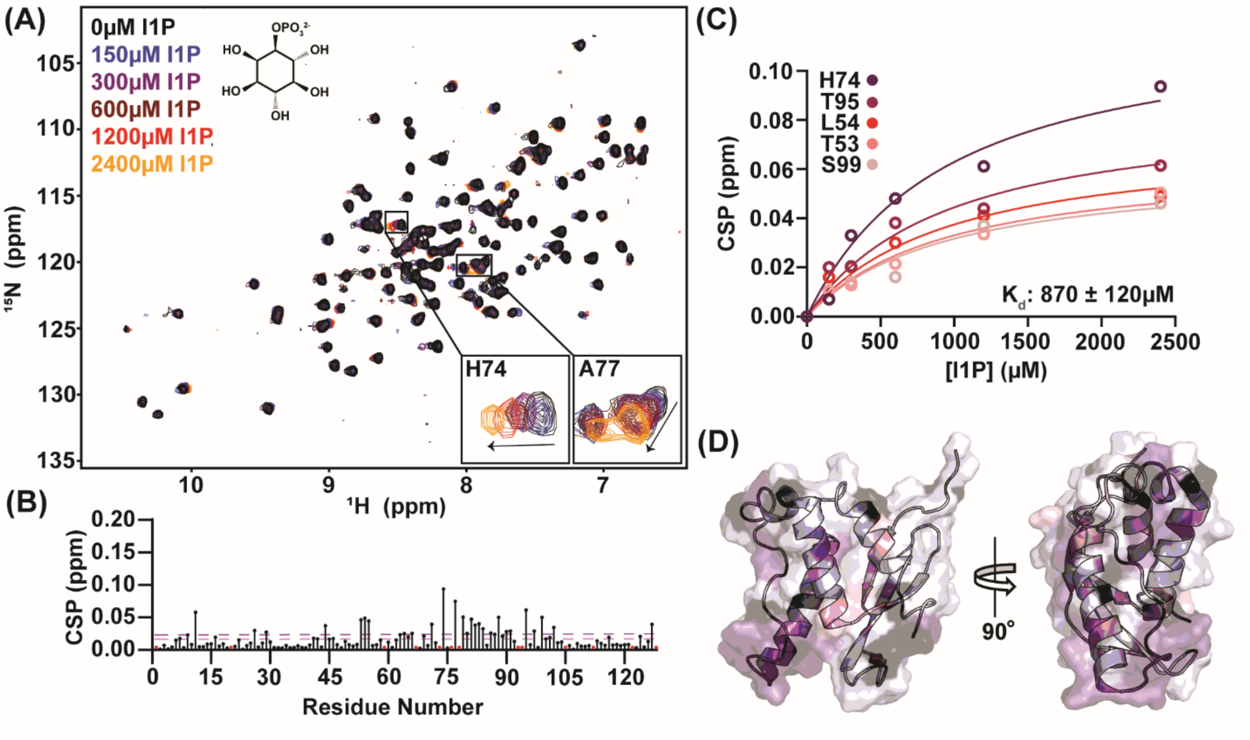

Figure S6. NMR titration of inositol-1-phosphate (I1P) (A) Spectral overlay of titration encompassing 0 – 2.4 mM I1P with 100 μM PX domain. (B) Chemical shift perturbations per residue of p47^{phox}-PX upon addition of 2.4 mM I1P. Pink and purple dashed lines represent 1 and 2 σ of a 20% trimmed mean, respectively. Unobservable residues are depicted as red circles offset from the x-axis. (C) K_d fitting of 2.4 mM I1P to p47^{phox}-PX. (D) 2.4 mM I1P CSPs mapped to the PX domain (PDB: 1GD5). Residues highlighted in pink and purple represent CSPs greater than 1 and 2 σ of a 20% trimmed mean, respectively. Unobservable residues are highlighted in black.

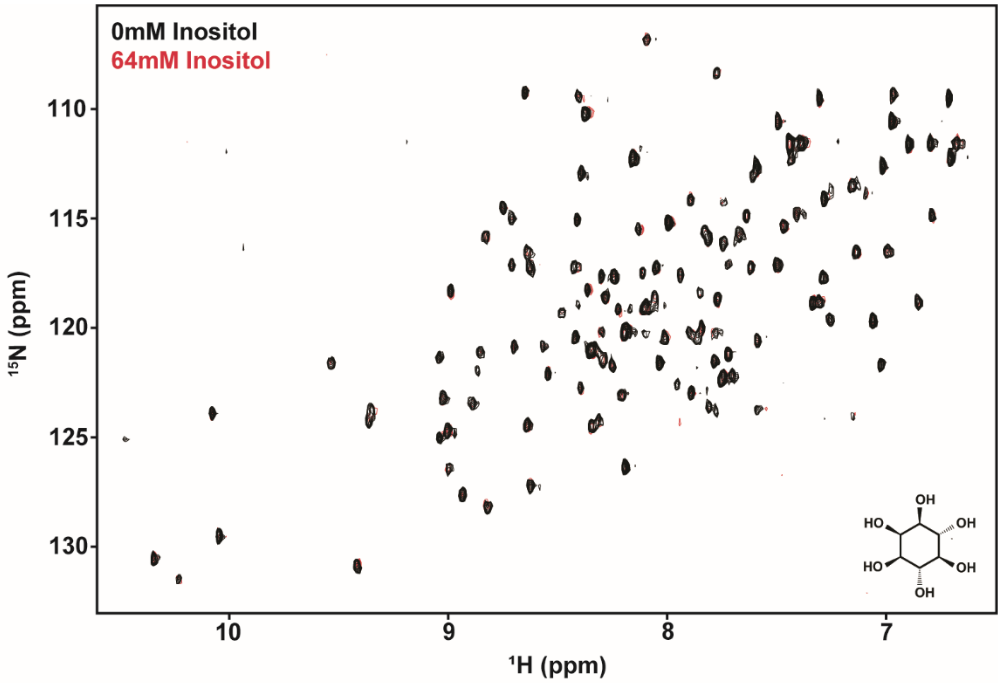

Figure S7. NMR spectral overlay of 100 µM p47^{phox}-PX with and without 64 mM inositol, depicting no significant binding in the absence of phosphorylated positions around the inositol ring.

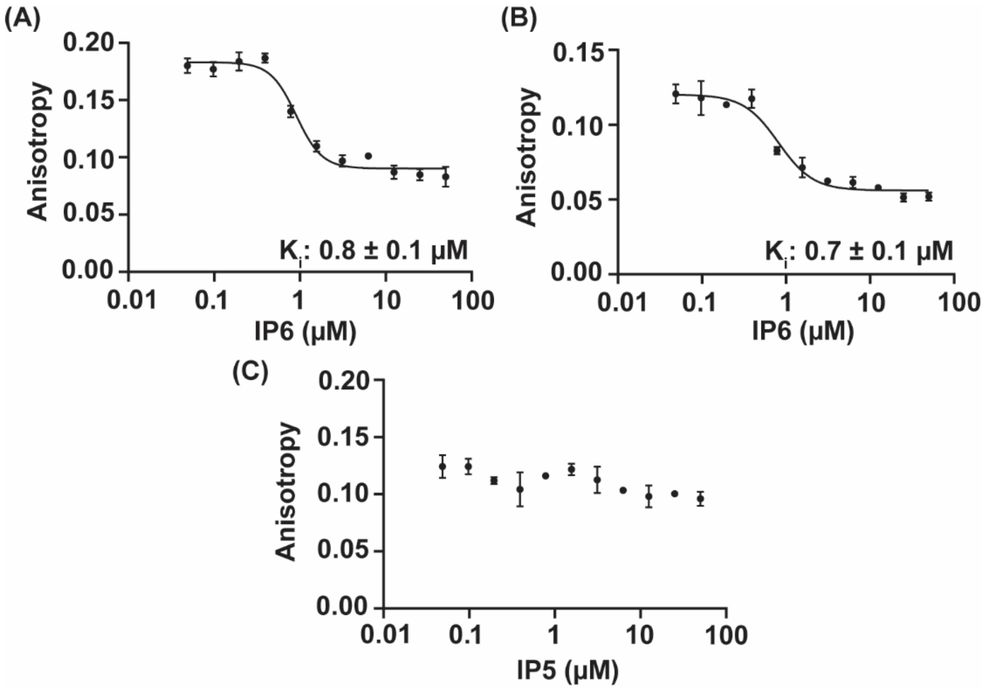

Figure S8. Fluorescence polarization competition measurements of IP5(1,3,4,5,6) and IP6 in low-pH or no $Mg2^+$ conditions. (A) FP titration of IP6 against BODIPY-PI(3,4)P_2 and p47^{phox}-PX at pH 6 with 1 mM $MgCl_2$ shows no significant change in K_i as compared to pH 7.0. (B) FP titration of IP6 against BODIPY-PI(3,4)P_2 and p47^{phox}-PX at pH 6 with no $MgCl_2$ shows no significant change in K_i in the absence of physiological $MgCl_2$ concentrations. (C) FP titration up to 50 μM IP5 showed no apparent capacity to inhibit the PX domain-BODIPY-PIP binding.
